# Nanofiber-Mâché Hollow Ball Mimicking the Three-Dimensional Structure of a Cyst

**DOI:** 10.3390/polym13142273

**Published:** 2021-07-11

**Authors:** Wan-Ying Huang, Norichika Hashimoto, Ryuhei Kitai, Shin-ichiro Suye, Satoshi Fujita

**Affiliations:** 1 Department of Advanced Interdisciplinary Science and Technology, Graduate School of Engineering, University of Fukui, 3-9-1 Bukyo, Fukui-shi 910-8507, Japan; ivy@biofiber-fukui.com (W.-Y.H.); suyeb10@u-fukui.ac.jp (S.-i.S.); 2 Department of Neurosurgery, Fukui Health Sciences University, 55-13-1 Egami, Fukui-shi 910-3190, Japan; hashi19721223@gmail.com; 3 Department of Neurosurgery, Kaga Medical Center, Ri 36 Sakumi, Kaga-shi 922-8522, Japan; rkitai@gmail.com; 4 Department of Frontier Fiber Technology and Science, University of Fukui, 3-9-1 Bukyo, Fukui-shi 910-8507, Japan; 5 Organization for Life Science Advancement Programs, University of Fukui, 3-9-1 Bukyo, Fukui-shi 910-8507, Japan

**Keywords:** electrospinning, nanofiber, hollow ball, alginate, tissue engineering, 3D structure

## Abstract

The occasional malignant transformation of intracranial epidermoid cysts into squamous cell carcinomas remains poorly understood; the development of an in vitro cyst model is urgently needed. For this purpose, we designed a hollow nanofiber sphere, the “nanofiber-mâché ball.” This hollow structure was fabricated by electrospinning nanofiber onto alginate hydrogel beads followed by dissolving the beads. A ball with approximately 230 mm^3^ inner volume provided a fibrous geometry mimicking the topography of the extracellular matrix. Two ducts located on opposite sides provided a route to exchange nutrients and waste. This resulted in a concentration gradient that induced oriented migration, in which seeded cells adhered randomly to the inner surface, formed a highly oriented structure, and then secreted a dense web of collagen fibrils. Circumferentially aligned fibers on the internal interface between the duct and hollow ball inhibited cells from migrating out of the interior, similar to a fish bottle trap. This structure helped to form an adepithelial layer on the inner surface. The novel nanofiber-mâché technique, using a millimeter-sized hollow fibrous scaffold, is excellently suited to investigating cyst physiology.

## 1. Introduction

Cysts are spherical sac-like multicellular structures with fluid and semisolid matter inside. Cysts occur in every kind of tissue; they are usually harmless and heal spontaneously, but occasionally transform into malignant tumors. For example, an intracranial epidermoid cyst sometimes undergoes a malignant transformation into an intracranial squamous-cell carcinoma, which has a poor prognosis. Treatment of malignancies is difficult, especially if the brain stem is extensively involved in the cyst [1,2]. Most investigations of cyst biology have involved in vivo experimental models; elucidating cellular behaviors in the cyst in vitro has proved difficult. Previously reported methods of preparing three-dimensional (3D) cellular structures include the hanging drop method [3], the formation of spheroids by cell non-adhesive multi-plate [4] or micropatterned-substrate [5] methods, and the use of cell sheets [6], bioprinting [7], and hydrogels [8] for forming 3D cell aggregates. However, cell aggregates prepared by these methods are cell-filled structures; it has been impossible to form a hollow structure such as a cyst.

The approach introduced in this study was inspired by the fabrication of papier-mâché dolls from balloons and paper. Our model cyst is a controlled nano-structure, the “nanofiber-mâché ball”—a hollow nanofiber sphere made of biodegradable polyester. We have focused on electrospinning as a promising technique to fabricate geometrically controlled nanofibers [9,10,11]. Electrospun nanofibers with high orientation were deposited on the surface of hydrogel beads of alginate, a natural hydrophilic and anionic polysaccharide commonly obtained from brown algae that exhibits good biocompatibility and biodegradability [12,13]. Alginate hydrogel has been extensively investigated for biomedical applications such as cell scaffolding and drug release [10,14,15,16]. Alginate can easily form a hydrogel in the presence of divalent cations such as calcium, while cross-linked alginic acid can be chelated by ethylenediaminetetraacetic acid (EDTA) for easy dissolution [17]. We chose poly(3-hydroxybutyrate-*co*-3-hydroxyhexanoate) (PHBH), a biocompatible and flexible polyester, as the matrix fiber [18,19,20]. We used electrospinning to fabricate nanofiber with a specific topography that mimics the extracellular matrix (ECM) for controlling cell fate. To our knowledge, this is the first report of fabrication of a millimeter-sized and geometrically controlled cyst-like structure.

## 2. Materials and Methods

### 2.1. Materials

PHBH (3HH = 10.6 mol%) was provided by Kaneka (Tokyo, Japan); sodium alginate (500 cps) was obtained from Nacalai Tesque (Kyoto, Japan); chloroform (CHCl_3_) and calcium chloride (CaCl_2_) were obtained from FUJIFILM Wako Pure Chemical (Osaka, Japan); EDTA was obtained from Thermo Fisher Scientific (Waltham, MA, USA). All other chemicals and reagents were of analytical grade and were used without further purification.

### 2.2. Fabrication of Nanofiber-Mâché Ball

**Scheme 1:**
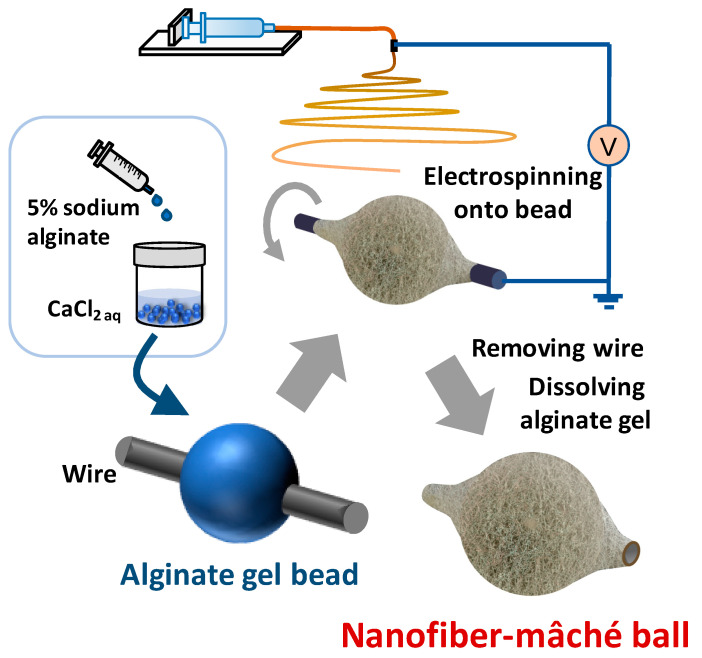
Conceptual illustration of the fabrication of nanofiber-mâché ball based on alginate gel beads.

The fabrication process is shown in Scheme 1. Alginate hydrogel beads (3 mm in diameter) were obtained by dropping 5 w/v% sodium alginate intermittently into 1 w/v% CaCl_2_. An aluminum wire (0.5 mm in diameter) penetrated the alginate beads and was then set up along the rotating axis of the electrospinning collector. PHBH solution (15 w/v% in CHCl_3_) was electrospun onto the alginate gel beads at 0.1 mL/h flow rate with 29 kV and a needle-tip–to–collector distance of 10 cm. The density and orientation were controlled by the spinning time and rotation speed of the collector, respectively. The innermost layer consisted of random fibers electrospun at the rotation speed of 100 rpm for 45 min; the intermediate layer of weakly aligned fibers electrospun at 500 rpm for 25 min; and the outermost layer of strongly aligned fibers electrospun at 1000 rpm for 25 min. After electrospinning, the alginate hydrogel gelled with calcium ions was washed with 10 mM EDTA solution six times for 10 min and kept in EDTA solution overnight to chelate the calcium ions. After washing and drying, the nanofiber-mâché ball was used in cell culture.

### 2.3. Morphological Observation

Morphological observation of the nanofiber-mâché structure was carried out using digital microscopy (VHX-2000, KEYENCE, Osaka, Japan) and scanning electron microscopy (SEM; JCM-6000Plus, JEOL, Tokyo, Japan). The fiber samples were immersed in liquid nitrogen and cut into several small pieces in a frozen condition.

For SEM observation, cells were fixed with fresh 4% paraformaldehyde for 30 min, washed, dehydrated with sequentially diluted ethanol, and then transferred into t-butyl alcohol for 2 h. Both fiber samples and cell samples were lyophilized with a freeze-dryer. The samples were sputtered with Os for 10 s using an ion sputter (HPC-1SW, Vacuum Device Inc., Ibaragi, Japan) and observed using SEM at an accelerating voltage of 15 kV.

To measure the diameter of nanofiber and collagen fibrils, 100 randomly selected fibers were counted using image-processing software (Fiji.sc; Ver 2.0.0, ImageJ, NIH, USA). The nanofiber orientation was quantified by the second-order parameter *S*, defined by

(1) $$S=2<\cos^{2}\theta >-1=<\cos2\theta >$$

where θ represents the orientation angle and <cos2θ> is the average of cos2θ [21,22].

### 2.4. Surface Characterization

Attenuated total reflectance-Fourier transform infrared (ATR-FTIR) spectrometry measurement was performed to verify the composition of nanofibers on a Nicolet 6700 system (Thermo Scientific, Waltham, MA, USA), with the wave number range from 500 cm*^−^*^1^ to 4000 cm*^−^*^1^ at a resolution of 4 cm*^−^*^1^ with an average of 64 scans. The hydrophilicity of the surface of nanofiber electrospun on a cover slip was measured using a water contact angle meter (DSA25E, KRÜSS, Hamburg, Germany).

### 2.5. Cell Culture with Nanofiber-Mâché Ball

To improve its surface hydrophilicity, the nanofiber-mâché ball was treated with oxygen plasma (40 kHz/100 W, 0.1 MPa, 30 s; Femto 9, Diener electronic, Ebhausen, Germany) and washed twice with 30% EtOH and PBS, then coated with 10 µg·mL^−1^ of fibronectin for 30 min at 37 °C. U-251 MG cells, derived from human glioblastoma astrocytoma, were cultured in high glucose Dulbecco’s modified Eagle’s medium (DMEM; Fujifilm Wako Pure Chemical Corporation, Osaka, Japan) supplemented with 10% fetal bovine serum (FBS; JRH Biosciences, Lenexa, KS, USA) and 1% Penicillin-Streptomycin solution (Fujifilm Wako Pure Chemical Corporation). Cell suspension, mixed with 3.75% methylcellulose (#400, Nacalai Tesque, Japan), was injected into the nanofiber-mâché ball at a density of 1 × 10^4^ cells/μL and incubated in a humidified atmosphere with 5% CO_2_ at 37 °C for 72 h.

### 2.6. Statistical Analysis

All values were expressed as mean and standard deviation. Student’s *t*-test was performed and *p* < 0.05 was considered statistically significant.

## 3. Results

### 3.1. Morphology of Nanofiber-Mâché Ball

**Figure 1:**
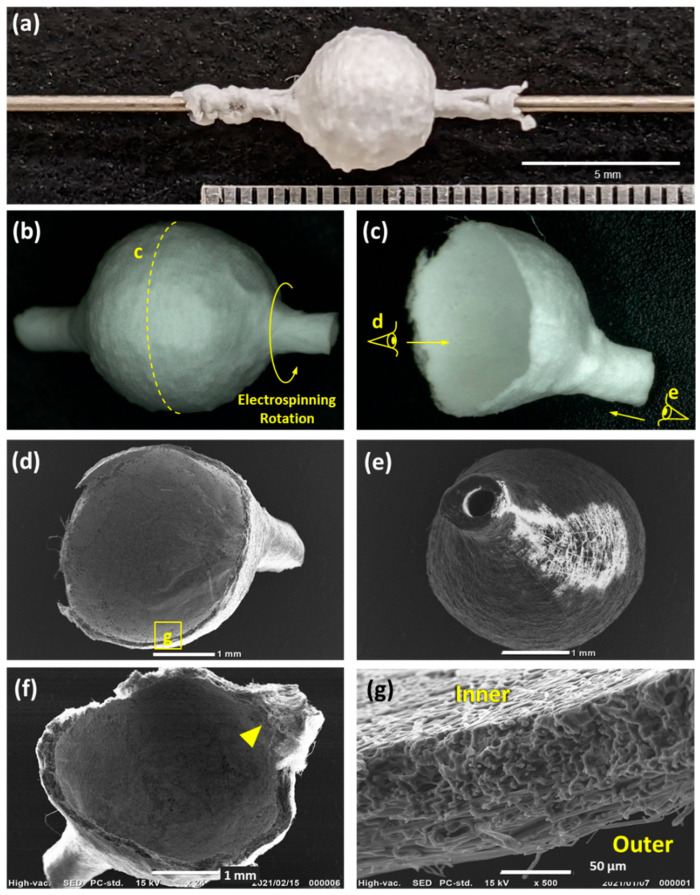
(**a**) Nanofiber-mâché ball electrospun on a wire. (**b**) Macroscopic pictures of overall structure and (**c**) nanofiber-mâché semi-sphere. (**d–g**) SEM images showing morphology of nanofiber-mâché ball. (**d**) Front view of semi-sphere, showing cup structure. (**e**) Back view of semi-sphere, showing a cell-seeding hole. (**f**) Longitudinal section of nanofiber-mâché ball and duct. (Arrowhead: interface between duct and central sphere.) (**g**) High-magnification cross-sectional view of nanofiber-mâché ball. The different fiber geometries and degrees of alignment on the inner and outer surfaces was observed.

PHBH fiber was electrospun on an alginate bead fixed on a wire (Figure 1a). To reveal the outer and inner structural morphology of the fabricated hollow nanofiber-mâché balls after the washing process, two semi-spheres were observed using digital microscopy (Figure 1b,c). The actual inner and outer diameters of a nanofiber-mâché ball were found to be approximately 3.8 and 4.1 mm, respectively. The interior of the ball, corresponding to the microenvironment where cells reside in tissues, had a volume of 230 mm^3^. The two ducts formed after the core aluminum wire was removed, located at opposite sides of the hollow ball, had inner diameters of approximately 0.58 mm; they can be used to access the interior for injection of cell suspension. Fine structures were observed in detail by SEM. In a sectional view of the nanofiber-mâché ball, it was evident that the inner and outer structures were distinct. The inner fibers were spun randomly to form a smooth surface (Figure 1d), whereas the outer fibers were spun along the circumferential direction (Figure 1e). Longitudinal-section observation of a nanofiber-mâché ball and duct showed details of the interface between the central sphere and duct on both sides (Figure 1f, arrowhead). The cross-section of the wall showed the geometric difference between the inner and outer surfaces (Figure 1g). The thickness of the inner layer was approximately 60.5 μm, while the outer layer was 53.9 μm in thickness. The total thickness of the wall was 114.4 μm.

### 3.2. Dimension and Geometry of Nanofiber

**Figure 2:**
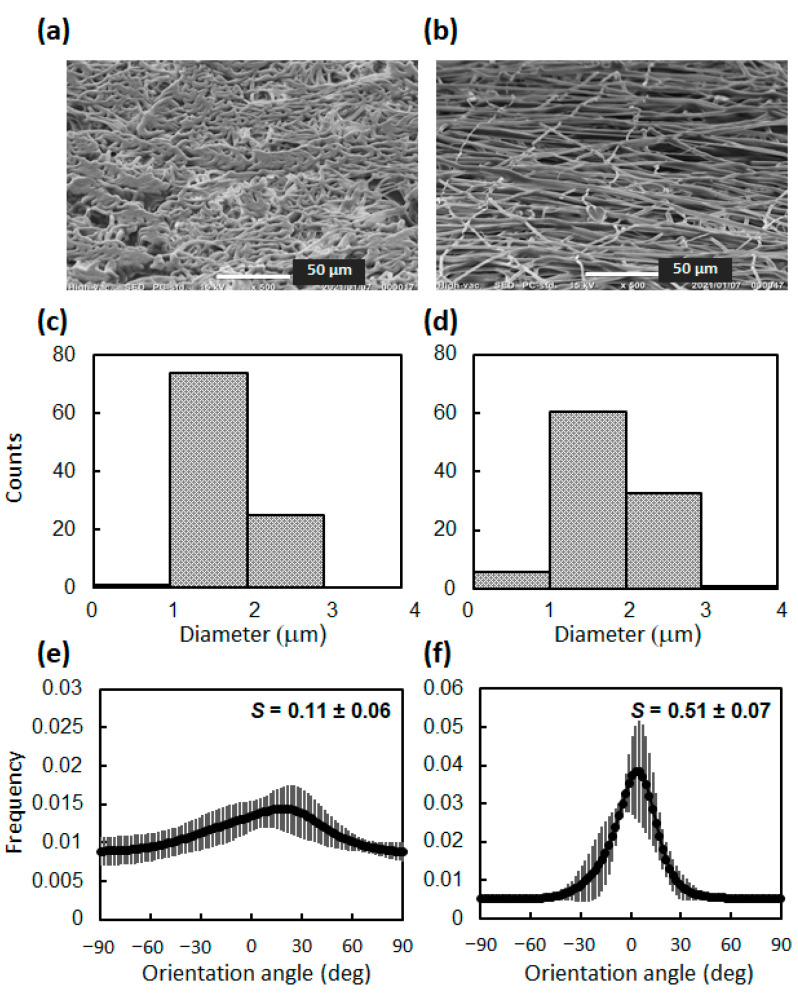
(**a,b**) SEM image, (**c,d**) fiber-diameter distribution, and (**e,f**) orientation distribution on the inner (**a,c,e**) and outer surface (**b,d,f**) of a nanofiber-mâché ball. The orientations of the inner (random) and outer (aligned) fibers were significantly different.

To reveal the dimension and geometry of electrospun nanofiber, the inner and outer surfaces were observed under high magnification (Figure 2a,b). The fibers on the inside were random; they were fused to each other as if they were pressed, because they were spun on the surface of alginate gel beads. The fibers were densely packed and adhered to each other, especially in the inner random region. Therefore, there was not enough space for cells to penetrate through the wall, although it was porous. This indicates that nutrients and oxygen can be supplied from the outside, but cells can migrate only through the duct.

The fiber diameter and secondary orientation parameters were measured from SEM images. The inner fibers were 1.70 ± 0.41 μm in diameter and had orientation parameter *S* = 0.11 ± 0.06 (Figure 2c,e). On the other hand, the fiber diameter and orientation parameter of the outer fibers were 1.76 ± 0.57 μm and 0.51 ± 0.07, respectively (Figure 2d,f). The size of the porous gap between the fibers was as same as the fiber diameter; we assumed that cells could move freely in such a space. We found within that fiber diameter no statistically significant changes when we altered the nanotopography of the nanofiber-mâché ball. It should be relatively easy to obtain balls of various types: orientation parameter is controllable by manipulating the size of the alginate beads and the rotation speed while electrospinning.

### 3.3. Characterization of Nanofiber Surface

**Figure 3:**
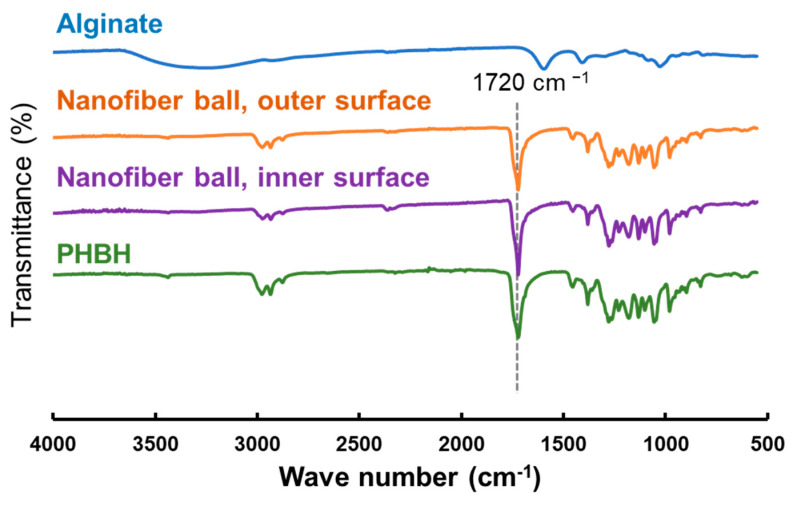
ATR-FTIR spectra of the alginate, inner, and outer surface of nanofiber-mâché ball, and PHBH.

ATR-FTIR spectra of PHBH, alginate, and the inner and outer surfaces of the nanofiber-mâché ball were measured (Figure 3). A specific peak of PHBH (1720 cm^−1^, C = O stretching) was observed in both inner and outer surfaces of the nanofiber-mâché ball, but no peak attributed to alginate was observed, which indicates that there was no residual alginate in the nanofiber-mâché ball after the dissolving process of alginate.

**Figure 4:**
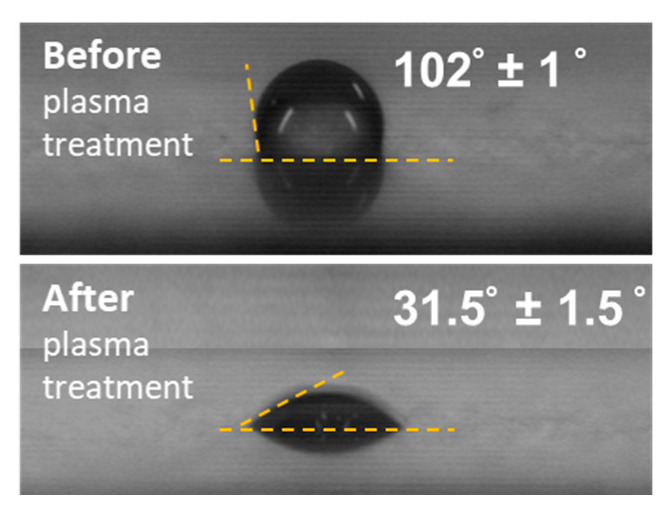
Water contact angle of electrospun PHBH nanofiber sheet before and after oxygen plasma treatment.

To show the improvement of surface hydrophilicity by oxygen plasma treatment, water contact angles of electrospun PHBH fiber sheets were measured by a sessile drop method (Figure 4). The water contact angles before and after plasma treatment were 102° ± 1° and 31.5° ± 1.5°, respectively. This result shows that oxygen plasma treatment hydrophilized PHBH, a hydrophobic polymer.

### 3.4. Cell Adhesion

**Figure 5:**
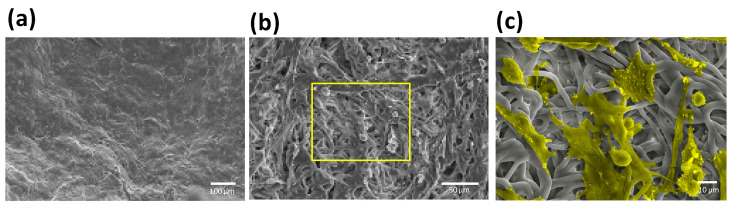
SEM images of U-251 MG culture on fibronectin coating nanofiber-mâché ball for 72 h. (**a**) Cells adhered to the inner surface, where the fibers were randomly oriented, at low magnification observation. (**b**) The adhesivity of uniform cells onto the inner surface (**c**) contributed to forming an adepithelial layer, at two magnifications. (Yellow in false-color image: cells).

While investigating the feasibility of cell culture in the nanofiber-mâché ball, we observed that U-251 MG cells (inoculated as a suspension mixed with methylcellulose medium) adhered to the whole fibronectin-coated inner surface of the ball (Figure 5a), the viscous medium having prevented them from settling. Interestingly, although the cells spread on the nanofibers, few penetrated beneath the tightly packed fibers (Figure 5b,c), so an adepithelial layer formed on the inner surface. This result indicates that the nanofiber mâché ball would provide a good microenvironment for cell adhesion.

### 3.5. Cell Orientation

**Figure 6:**
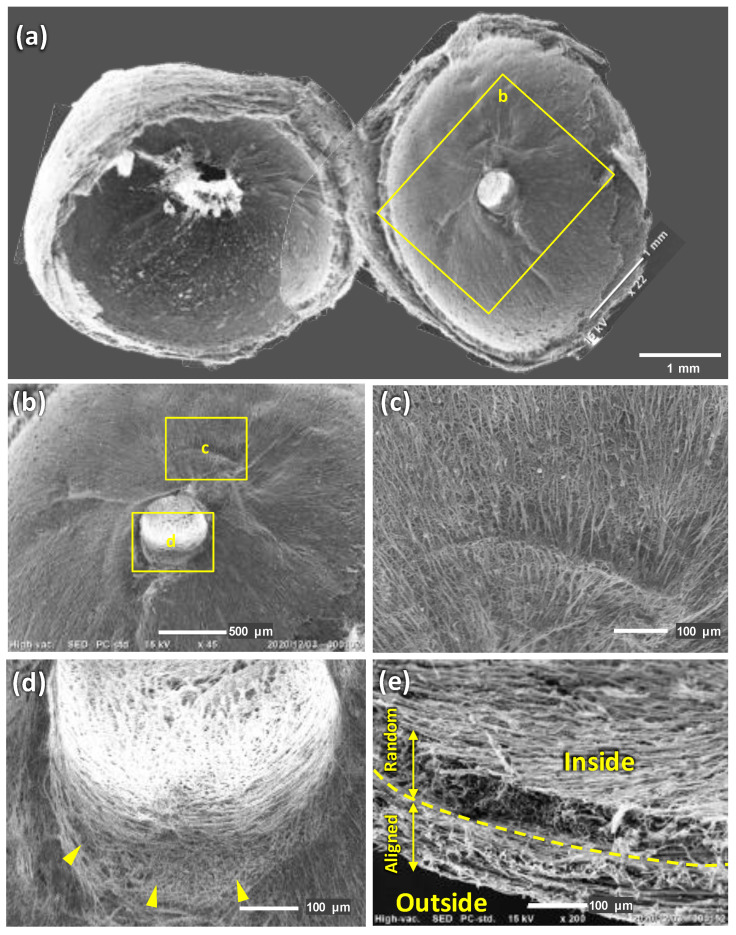
U-251 MG adhered to the inner surface of the nanofiber-mâché ball after a three-week culture. (**a**) Observation of the structure of the hollow nanofiber-mâché ball cut in half. (**b**) A cell layer tightly covered the inner surface after long-term culture. (**c**) The gradient in the concentration of the medium promoted cell alignment towards the duct exit. (**d**) Enlarged view of duct entrance. Cells oriented along aligned in the circumferential direction between the central sphere and ducts, but the periphery of the duct showed random towards the duct. (Curved arrow: circumferential fiber at the interface; arrowheads: fiber orientations on the periphery of the duct). (**e**) Invasion of cells (cross-sectional view). A layer of cells was covering the inner surface showed no obvious invasion into fibers.

To investigate the production of ECM in long-term cell culture, U-251 MG cells were inoculated into a ball that had been treated with oxygen plasma but not coated with fibronectin. In the seeding, cells were suspended in the medium without methylcellulose. After three weeks of culture, the ball was cut for SEM observation (Figure 6a). Cells had proliferated to cover the inner surface confluently (Figure 6b). As shown in Figure 6c, the cells formed highly orientated structures and were elongated in the radial direction toward the center of the duct. The reason that the cells aligned even though the inner surface consisted of random-spun fibers was the difference in the local concentration of nutrients and oxygen in the nanofiber-mâché ball. Each ball had two opposed ducts allowing access by the external medium with its nutrients and oxygen. The gradient in the concentration is a possible attractant for cell spreading and migration.

However, the cells near the periphery of the duct appeared to be randomly directed rather than aligned towards the duct (Figure 6d, arrowheads). It was also found that, at the sphere-duct interface itself, the fibers were aligned along the circumference of the duct (Figure 6d, curved arrow). This orientation of fibers was the result of electrospinning directly onto a conductive wire, not an alginate bead. When cells migrating out to the duct from the inner surface reached the transitional region, where the interfacial fibers aligned in the direction of the circumference, they were trapped and spread over randomly because the fiber orientation was perpendicular to the direction of migration. This fiber structure would work as a barrier to inhibit cells from migrating toward the outside, such as the one-way structure of a fish bottle trap.

The cross-section of the walls after long-term culture revealed the extent of cellular invasion (Figure 6e). Although cells in a layer covered the inner surface, they did not penetrate into the fibers deeply. The walls were non-porous and dense enough to inhibit cell migration. This observation indicates that the nanofiber-mâché ball could provide an adequate cell-adhesive surface.

### 3.6. ECM Production

**Figure 7:**
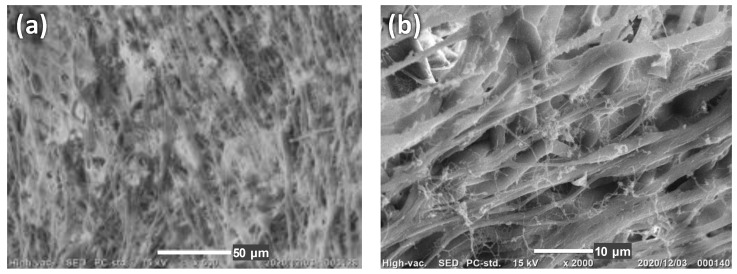
(**a**) High-density extracellular matrix (**b**) secreted web of collagen fibrils from U251 MG cells in a nanofiber-mâché ball.

High-magnification observation also revealed the production of a dense network of extracellular matrix structures (Figure 7a). Although the pre-fibronectin coating was not carried out in this culture, thin fibers with diameters 225 ± 136 nm were observed; they were much thinner than the PHBH scaffold and the cell itself (Figure 7b). Because they were similar in size to collagen fibrils, we consider them to be ECM fibers produced by U-251 MG cells.

## 4. Discussion

The 3D structures of intracranial epidermoid cysts consist of a thin outer layer of fluid-filled epithelial cells, keratin, and cholesterol. Establishing controllable-scale in vitro models of cysts will make our knowledge of pathogenesis more sophisticated, with prospective implications for optimizing the diagnosis of craniofacial dermoid cysts. There are two types of cutaneous cysts: dermoid, with epithelial cell walls containing either stratified or unstratified squamous epithelium, and periapical, encompassed by connective or granulation tissue. The former are often detected during infancy and early childhood, superficially or in the anterior orbit [23]. Their clinical severity, including the possibility of rupture and intracranial extension, may be directly affected by size and histologic differences [24]. In some clinical cases, craniofacial dermoid cysts of the frontotemporal showed subcutaneous sizes smaller than 15 mm × 15 mm in children [25]. At the other extreme, colloid cysts due to certain anatomical transmutations of the forniceal structures may have abnormally large sizes (>30 mm), and in an advanced stage of growth, a retro- or post-foraminal colloid cyst may separate from the forniceal structures if it is situated in the cavum septi pellucidi or vergae [26]. To determine the origin of cysts precisely from their sizes and histologic differences and predict the malignant possibility of lesions is a great challenge.

The nanofiber-mâché ball we developed is helpful for mimicking biological processes, in which the geometry and dimensions of 3D structures play key regulatory roles. The fiber orientation around the duct traps the cells inside the ball and prevents them from migrating out (Figure 6); the duct allows only fluid to pass between the interior and exterior. The narrow diameter of the duct also might induce small vortical flow, with cells orienting themselves along the direction of flow during culture [27]. This structure is a valid in vitro model of a fluid-filled cyst that is able to support tissues in vivo for oxygenation, nutrients, and waste removal.

The nanofiber-mâché ball with complex topography also provides efficient production of ECM network structure inside after long-term cell culture (Figure 7). The ECM is a composite network of the secreted products of resident cells containing structural and functional proteins that assemble in unique tissue-specific architecture; it serves as the scaffolding for tissues and organs in the body [28]. The microenvironment for tissue is supplied by ECM [29], including structural proteins such as collagen, cell-adhesion molecules such as fibronectin and laminin, and proteoglycans, which provide cells with a reservoir for biological signal molecules. Cells grow naturally in this 3D microenvironment that allows cell-cell and cell-matrix interaction and provides efficient biological signaling. Cell culture on a 3D model gives rise to cell interactions because its spatial packing influences a series of cellular behaviors, including cell morphology, proliferation, differentiation, the expression of genes and proteins, and cellular responses to extrinsic stimulation [30]. It can be used for the further investigation of the biological role of ECM in cysts.

Our model also has the potential to develop the optimal therapeutic strategy of a giant thrombotic aneurysm, which is a type of proliferative cerebral aneurysms because it absorbs nutrients from the outer blood vessel wall even if coil embolization or parent vessel occlusion treatment was performed at the inside of the aneurysm [31,32]. The nanofiber-mâché ball model can be used for the analysis of the growing mechanism of thrombosed aneurysms characterized by organized intraluminal thrombus and solid mass. Moreover, we expect that the bioprinting of alginate hydrogel has been rapidly advancing and it would be also applicable to prepare templates for nanofiber-mâché mimicking other tissues and organs.

## 5. Conclusions

We have successfully created a novel 3D hollow nanofiber-mâché ball by dissolving alginate beads. The hollow spaces left by the beads and the anisotropy of the nanofibers mimic the environment of cysts for cell-culture models. PHBH hollow nanofiber-mâché balls provide an in vitro culture system for the study of cell-cell and cell-ECM interactions. Our experimental model facilitates the analysis of biological behavior inside the cyst and may eventually shed light on phenomena that have not been fully elucidated clinically, such as the malignant transformation from epidermoid cysts to squamous cell carcinomas. Although further research is still needed, this preliminary study suggests great potential for medical applications.

## Data Availability

The raw/processed data required to reproduce these findings cannot be shared at this time due to legal reasons.
